# Knowledge, Beliefs, and Perceptions of TB and Its Treatment amongst TB Patients in the Limpopo Province, South Africa

**DOI:** 10.3390/ijerph181910404

**Published:** 2021-10-02

**Authors:** Hulisani Matakanye, Takalani Grace Tshitangano, Jabu Tsakani Mabunda, Thelmah Xavela Maluleke

**Affiliations:** 1 Department of Public Health, School of Health Sciences, University of Venda, Thohoyandou 0950, South Africa; Takalani.Tshitangano@univen.ac.za (T.G.T.); Jabu.Mabunda@univen.ac.za (J.T.M.); 2 Department of Public Health, School of Health Care Sciences, University of Limpopo, Sovenga 0727, South Africa; thelmah.maluleke@gmail.com

**Keywords:** knowledge, beliefs, patients, perceptions, tuberculosis, treatment

## Abstract

Despite the implementation of global plans to end tuberculosis (TB), this disease remains a major cause of ill-health and is the second leading cause of death. In 2019, globally, an estimated 10.0 million people fell ill and about 1.4 million died of TB. During the same year, South Africa was among the eight countries with the highest tuberculosis burden, contributing to two-thirds of the world’s total. Due to the high burden of the human immunodeficiency virus (HIV) epidemic, TB in South Africa is a major public health problem. Limpopo is amongst the provinces that are hardest hit by TB and HIV in South Africa. Therefore, using a quantitative descriptive design, this study assessed the knowledge, beliefs, and perceptions of TB and TB treatment amongst TB patients in the Limpopo Province. A systematic random sampling method was used to sample 207 TB patients who agreed in writing to be part of the study. Data were collected using a self-administered questionnaire, however, about 3.9% of participants who could not read were assisted by the main researcher and health professional. About 33% of the participants with primary education were also assisted to clarify any questions that were not clear to them. Data were analyzed using the Statistical Package for Social Sciences^®^ version 26.0. Validity and reliability of the instrument was ensured, and ethical considerations were observed in this study. The research findings revealed that about 93.25% respondents only came to know they had TB after diagnosis. About 75% indicated that they had visited faith healers and only 2% acknowledged that they had visited traditional healers after diagnosis. A total of 76% of the respondents stated that they strongly believed in their culture and religion. These findings highlight the need for health education efforts to strengthen accurate information to improve TB knowledge and correct misconceptions about TB among patients within the community.

## 1. Introduction

Tuberculosis (TB) is a major global health problem, infecting millions of people annually. TB ranks alongside the human immunodeficiency virus (HIV) as a leading cause of death worldwide, particularly in low- and middle-income countries [1]. Despite the availability of efficacious treatment for TB, high morbidity, and mortality are observed. In 2017, TB caused about 1.6 million deaths globally. In 2019, there were an estimated 10 million new TB cases worldwide, of which 5.6 million were men, 3.2 million were women, and 1.2 million were children. People living with HIV accounted for 10% of the total. It is estimated that about one-third of the world’s population is infected with the tuberculosis bacillus [2]. In 2015, the highest number of new TB cases occurred in Asia (61%), followed by Africa with 26% of new cases and usually infects adults in their productive years [3]. TB has for many years been the leading cause of deaths from a single pathogen, however, it is now the second leading cause of deaths worldwide due to the rise of severe acute respiratory syndrome coronavirus 2 (SARS-CoV-2) [4].

In 2017, about 72% TB patients were in Africa and two thirds were from the following eight countries: India (27%), China (9%), Indonesia (8%), the Philippines (6%), Pakistan (5%), Nigeria (4%), Bangladesh (4%), and South Africa (3%) [3]. These eight countries and 22 other countries in the WHO’s list of 30 high TB burden countries accounted for 87% of the world’s cases. Significantly, Europe and the Americas reported only 3% each of the TB global cases. This shows that the severity of national epidemics varies widely among countries. In 2017, there were fewer than 10 new cases per 100,000 population in most high-income countries, 150–400 in most of the 30 high TB burden countries, and above 500 in countries such as Mozambique, the Philippines, and South Africa [3].

TB continues to be the second leading cause of death after SARS-CoV-2 in South Africa. In 2019, 58,000 people died of TB and of these, about 36,000 were HIV positive [2]. The burden of TB in South Africa is fueled mostly by the high HIV pandemic. Limpopo is one of the provinces most affected by TB with approximately 55% of TB patients in the province co-infected with HIV. Only 76% of adults know their status compared to 70% of children under 15 years living with HIV [5]. In some regions of Africa, about 75% of TB patients are co-infected with HIV [6]. People living with HIV are at a higher risk of developing TB than those who are not HIV-infected. According to Health System Trust, in 2016, the Limpopo Province had the highest number of patients at 96.7% initiated on TB treatment. The province had a treatment cure rate of 76.1% compared to the national target of 85% [7], and had a TB treatment defaulter rate of 6.1% compared to the national target of 5.4%. While the TB treatment success rate was 80.6%, this was below the national target of 85% and the TB death rate was 11.5% compared to the national average of 5% [7].

Lack of knowledge about the TB disease causes an underutilization of the health services, delay in seeking diagnosis, and poor treatment adherence amongst TB patients. Improving the roll out of TB public awareness and health promotion is important in increasing knowledge about TB. This, in turn, shapes the community and TB patients’ health-seeking behavior [8]. Different studies have reported that non-adherence to treatment often results from inadequate knowledge or understanding of the disease and its treatment [9]. Although people have a general idea of what TB is, there are gaps in knowledge on transmission, treatment, and prevention. This leads to diagnostic and treatment delays for people living with TB [10]. Another study indicated that patients with low TB knowledge are less likely to seek health care and get diagnosed. Instead, they often turn to self-medication and traditional healers, which lead to delays in diagnosis and appropriate treatment [8]. In Africa, it has also been reported that community members often have incorrect knowledge about the cause and transmission of TB [11].

Poor understanding of TB and TB treatment leads to non-adherence to TB treatment [12]. Non-adherence to the treatment results in uncured TB, spreading of TB in communities, and an increase in TB drug resistance. Patients with drug resistant TB often develop TB that is resistant to at least one first-line anti-TB drug. If the patient continues to be non-adherent to treatment, the patient may develop multidrug-resistant TB (MDR-TB). Drug-resistant TB requires close management and consultation with experts in the disease [13]. According to the WHO, non-adherence to TB treatment among patients is widespread and results in high mortality rates, the occurrence of drug-resistant TB, and increased treatment cost. The implementation of DOTS by WHO globally was an attempt at reach 100% adherence to treatment by TB patients, which in turn was expected to increase cure rates [14].

## 2. Methods 

### 2.1. Study Design

The study used a quantitative descriptive design to assess the knowledge, beliefs, and perceptions of TB and TB treatment amongst TB patients in Limpopo Province. Quantitative research methods were chosen, and numerical data were collected from a small group of people. The results were then generalized to a larger group to explain the study phenomenon. Quantitative research helped in ensuring that a suitable sample size was used to gain accurate and trustworthy results.

### 2.2. Study Site

The study was conducted in the Limpopo Province in the three selected districts of Waterberg, Capricorn, and Vhembe. According to the Health System Trust [7], the province has a population of 5,630,467 divided into 2,649,115 of males and 2,981,352 females. Approximately 80% of the population in Limpopo live in rural areas. The province experiences high numbers of both legal and illegal immigrants. The traditional health system is strong, and communities are influenced by their cultures and beliefs. This has a strong influence on TB treatment non-adherence and hence the reason for conducting this research in the province. 

### 2.3. Study Population

The study population was defined as all TB patients older than 18 years who had completed at least one month of TB treatment and were registered in the selected Community Health Centers (CHCs) in the Limpopo Province during the time of data collection. TB patients who were below the age of 18 were not included in the study.

#### Inclusion and Exclusion Criteria

All TB patients who were above the age of 18 and had completed at least one month of TB treatment and registered in the selected CHC TB register were included in the study. Respondents who had already completed their TB treatment from the date of data collection were excluded in the study. 

### 2.4. Sampling Procedure

The researcher used systematic random sampling to select samples at a preset interval. The sampling of the respondents was determined through Slovin’s formula and depended on the total number of TB patients registered on the TB register of CHCs. N was the total number of TB patients who were registered in the TB register of the selected CHCs during the time of the study and *n* was the sample size. The propositional sampling sizes were calculated based on the total population of CHCs. A sample size of 292 was determined. The total number of the TB registered patients (N = 1075) were then divided by the sample size (*n* = 292) to find the following interval Kth value: Kth value = 1075/292 = 3.68, which was 4. Systematic random sampling technique was then employed to sample TB patients from the CHCs’ TB registers, in order to sample every fourth TB patient for participation in the study. The researcher then randomly chose the starting number to be patient number one (1) who met the criteria as it appeared in the TB register until the total of 292 was reached. The researcher then added about 10% (28) to cover for non-response and the sample became 320 and was proportionally distributed. Subsequently, only 207 respondents participated in the study since others were not available due to COVID-19 lockdown. Most participants responded to the questionnaire from their homes.

### 2.5. Sample Size Determination

Slovin’s formula, where N is the total number of TB patients registered in the TB register of the selected CHCs during the time of the study, *n* is the sample size, and e is the accepted level of error, was used to calculate the sample size. The accepted level of error, e, was 0.05. The estimated overall sample size was 292 of all TB patients drawn from an estimated total population of 1075, with a 95% confident interval.

(1) $$n=\frac{N}{1+Ne^{2}}=\frac{1075}{1+\left(1075\times 0.05^{2}\right)}=\frac{1075}{1+\left(1075\times 0.0025\right)}=\frac{1075}{1+2.68}=\frac{1075}{3.68}=292$$

### 2.6. Data Collection Instrument

Data were collected using a self-administered questionnaire (Supplementary Material File) with open and closed-ended questions. The English language written questionnaire was developed by the researcher and reviewed and approved by the study promoters. The questionnaire was divided into four sections. Section A was socio-demographic characteristics that included age, employment status, sex, income, type of income, and level of education; Section B required knowledge about TB and TB treatment; Section C required participants’ information about the cultural, religious, and traditional beliefs regarding TB and TB treatment; and Section D sought to collect information relating to the participants’ perception of TB and its treatment. Section A had eight main questions, Section B had 14 main questions, Section C had eight main questions, and Section D had four main questions. The construct of the questions was open and closed-ended questions, which had two to six answers to choose from. The researcher ensured that all relevant topics were covered. The research participants freely responded to open and closed-ended questions and all unclear questions were explained in more detail to the respondents whenever they required clarity.

### 2.7. Data Collection Procedure

Data collection started after ethical clearance and permission to enter the CHCs were obtained. Data collection took place from June 2020–September 2020. Health care facility managers were engaged, and the details of the study were explained to them. Arrangements to call the selected respondents who met the criteria to come to the facility were made with the facility managers. Appointments were then made with the respondents and the researcher visited the facility on the day of the appointment. Those who could not make it to the facilities were visited at their homes. Respondents were asked to sign consent forms prior to participation. The researcher explained the purpose of the study to each participant before completing the questionnaire. Participants were assured that confidentiality would be maintained. All respondents who agreed to sign a written consent forms were included in the study. About 207 respondents who signed consent forms were given questionnaires to complete. About 3.9% of those who could not read were assisted by the main researcher and health professionals working at the CHCs. About 33% of the participants with primary education were also assisted to clarify any questions that were not clear to them. Questionnaires were collected the same day after the participants completed them. About 207 questionnaires were received from the respondents who were available during the time of data collection. With the help of the clinicians, the response rate was 71% as other patients were not available during the time of the study due to COVID-19 restrictions. Some patients had already been transferred from the facilities and were not from the surrounding villages. Only respondents from the surrounding areas were followed home and invited to participate in the study.

### 2.8. Data Analysis

Quantitative data analysis was carried out using Statistical Package for Social Sciences (SPSS) version 26.0 (2017). All data from the questionnaire were coded using a code book and was entered into SPSS version 26.0 statistical programs. The researcher used codes rather than the respondents’ names and checked the data by frequency to identify missing or incorrect values. In this respect, the mean, mode, median, and standard deviation variables were used to calculate the various descriptive statistics. Results emanating from the analysis were represented in the form of tables and charts.

### 2.9. Validity of the Instrument

Validity relates to the degree to which the research measures what it is supposed to measure [15]. The researcher focused on the content validity and face validity of the instrument.

#### 2.9.1. Face Validity

Face validity of an instrument refers to the judgment that an instrument is measuring what it is supposed to, based primarily on the local link between the questions and study objectives. The questionnaire was presented at the departmental seminars and School of Health Sciences’ Higher Degrees Committee at the University of Venda. The supervisors were consulted for comment and opinion as to whether the instrument would be suitable for collecting adequate data to develop an effective intervention for improving TB medical adherence. The questionnaire was restructured based on the feedback from supervisors.

#### 2.9.2. Content Validity

The researcher reviewed the literature and instruments from similar studies. Supervisors, TB coordinators, and experts in the field were consulted for comments and inputs before finalizing the instrument [16]. In addition, content validity was addressed by an extensive literature search to identify the domain of the construct before developing the questionnaire. 

### 2.10. Reliability of the Instrument

To ensure reliability of the instrument, the test re-test method was used, re-administering the same instrument to the same set of respondents. Ten percent (*n* = 30 respondents) of the sample size was sampled on day one, and the same set of respondents were given the same questionnaire two days later and the two responses were compared using the Cronbach Alpha correlation technique. A correlation co-efficient was used to compare the two instruments and a score was made on both instruments. A correlation co-efficient of 0.9 was found. This was closer to one (1), indicating a strong positive relationship and showed the reliability of the instrument [17].

### 2.11. Ethical Consideration

The proposal was submitted and presented to the School of Health Science and the University Higher Degrees Committee (UHDC), and an ethical clearance was granted (SHS/19/PH/28/0411). Permission to conduct the study was obtained from the Limpopo Provincial Department of Health and Vhembe District, Waterberg District, and Capricorn District Department of Health. Furthermore, permission was obtained from the facilities’ operational managers. 

All participants were asked to complete consent forms. The nature of the research was described to the respondents, and they were informed of their right to refuse to participate, or to withdraw from participating if they felt that they could not continue. The respondents were also informed and assured that the information within their responses would not be used against them or shared with other people, but would only be reported as study findings.

Anonymity was also ensured as respondents did not write down their names or any personal identification. The researcher respected the choices and agreements made with the respondents. The initial agreement was not changed without the knowledge of the respondents. Individuals were not victimized for refusing to participate. 

## 3. Results

From the selected cases of TB patients, there were 207 (71%) completed questionnaires out of the 292 targeted sample. This low response rate can be explained by the fact that the study took place during COVID-19 restrictions, and therefore some TB patients were not available. The results are presented below through the following categories: demographic profile of the respondents, knowledge about TB and TB treatment, beliefs about TB and TB treatment, and perception of TB and TB treatment.

### 3.1. Demographic Profile of the Respondents

The ages of respondents ranged from 18 to over 60 years. The frequency and percentage differed with age. The data showed that about 42% of the respondents were between the ages of 18 and 29. Only 5.3% of respondents were over the age of 60 and this could be because some patients were not willing to participate in the study due to the fear of COVID-19. All respondents were Africans, and the majority (84.1%) were born in South Africa where they still reside, while only 15.9% were born in Zimbabwe. Only 16.4% were married, 45.4% were single, 25.6% were living with partners, and 12.6% were widowed. A total of 3.9% said they had no schooling, 33.3% had only primary school education, 47.3% had secondary school education, and 15.5% had tertiary level education. About 70.0% of the respondents indicated that they were unemployed, 18.4% self-employed, and only 11.6% were formally employed. The majority (65.7%) of respondents indicated that they did not have any income, about 27.5% had a monthly income of between R400 and R3000, 4.3% had a monthly income of between R3500 and R15000, and only 2.4% had a monthly income of R16000 and above. About 93.7% respondents were from a Christian background, 2.4% subscribed to an African (Ancestors) religion, and 3.9% indicated that they did not belong to any religion (see Table 1). 

### 3.2. Knowledge about TB and TB Treatment

#### 3.2.1. Respondents’ Awareness of Their TB Status before Diagnosis

The study explored whether respondents were aware that they could have TB when they were sick. A total of 193 (93.2%) of the respondents confirmed that they were not aware that they had TB before a diagnosis. A total of seven (3.4%) suspected that they had TB even before diagnosis because they had symptoms consistent with the disease infection. A total of 3.4% respondents indicated they had TB because of the symptoms they were showing. 

#### 3.2.2. Respondents’ First Time of Learning about TB

**Figure 1:**
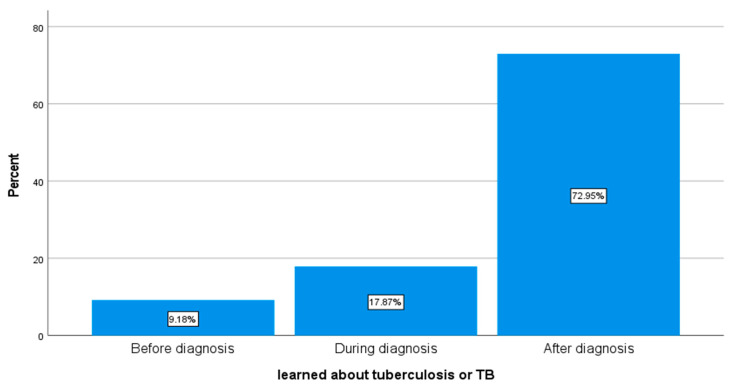
Respondents’ first time of learning about TB (*n* = 207).

In response to the question on when was the first-time the respondents heard or learned about TB, most (72.9%) respondents said that they had learned about TB after diagnosis, 17.9% had learned about TB during diagnosis, and only 9.2% had learned about TB before diagnosis (see Figure 1 below for details).

#### 3.2.3. Knowledge about the Cause of TB

All 207 respondents answered the question regarding the cause of TB. The following instruction was given on the questionnaire; only one response was allowed per question unless another instruction was given: “Thus, we do not really know the belief of the respondents regarding the cause of TB”. All we know is that when they were asked to select among the three possible answers of “bacteria, evil eye or witchcraft, and don’t know”, about 74% chose bacteria, none chose witchcraft, and about 26% chose “I don’t know”.

#### 3.2.4. Knowledge of the Transmission of TB 

**Figure 2:**
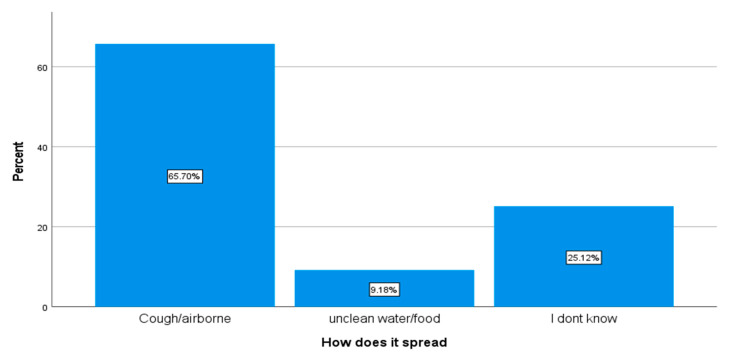
Knowledge about transmission of TB (*n* = 207).

Sixty five percent (65.7%) of the respondents indicated that TB spread through coughing and that it is an airborne disease, 9.2% said TB is spread through unclean food and water, and 25.1% admitted that they did not have any idea about the transmission of TB (see Figure 2).

#### 3.2.5. TB Can Be Cured If Treated Correctly

All 207 respondents answered the question that sought to understand whether they knew whether TB could be cured if it is treated correctly when TB treatment is taken for the correct duration of time which is 6–8 months. Respondents were asked to choose either “Yes or No”, where about 97.58% said TB could be cured if treated correctly, while only 2.42% indicated that TB could not be cured even if treated correctly.

#### 3.2.6. TB Can Result in Death If Not Treated

Respondents were asked whether TB could result in death if not treated, and they were asked to choose one out of the following three answers: “Yes, No, or Don’t know”. About 97.6% said that TB could result in death if not treated, only 2.4% said that they were uncertain about the consequences of untreated TB, and none of the respondents chose no.

#### 3.2.7. Importance of Completing TB Treatment

**Figure 3:**
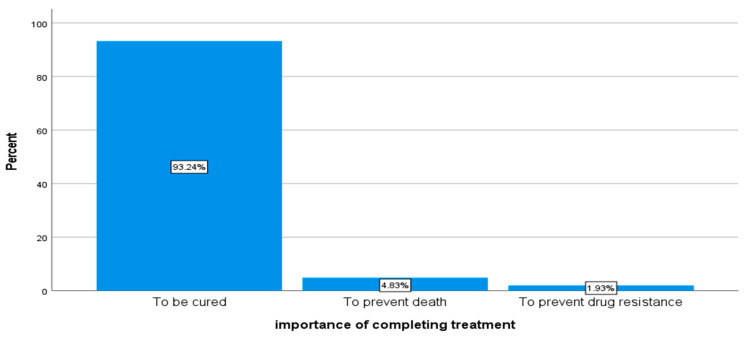
Importance of completing TB treatment (*n* = 207).

Respondents were asked if it is important to complete TB treatment and 93.2% indicated that it is important for one to be cured, 4.8% indicated that it is important in preventing death from TB, and 1.9% said it is important in preventing drug resistance (see Figure 3).

#### 3.2.8. Anti-TB Drug Side Effects

**Figure 4:**
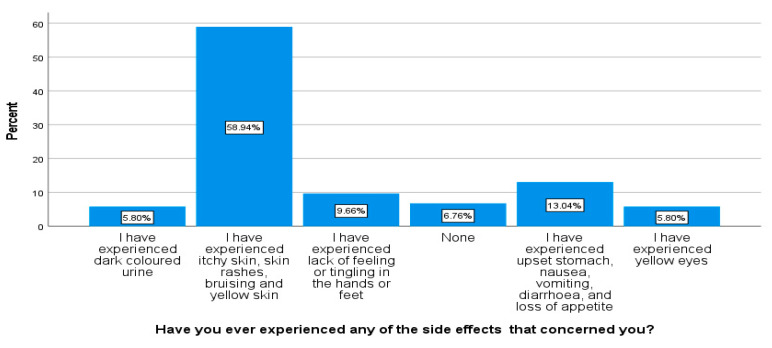
Review of whether respondents had ever experienced any TB treatment side effects (*n* = 207).

Respondents were asked if they had ever experienced any treatment side effects and most answered in the affirmative. A total of 58.9% had experienced itchy skin, skin rashes, bruising, and yellow skin. A total of 13.4% had experienced upset stomach, nausea, vomiting, diarrhea, and loss of appetite. About 9.6% had experienced lack of feeling or tingling in the hands or feet. A total of 5.8% had experienced yellow eyes and 5.8% had experienced dark colored urine. Only 6.7% said that they had not experienced any side effects (see Figure 4).

#### 3.2.9. Informed and Educated on Anti-TB Drug Side Effects 

The study further explored whether respondents were told what to do if they experienced any of the treatment side effects. The study listed the following answers that respondents had to choose from: “Yes, No, or Only when I had experienced them”. A total of 72.9% respondents said that they were informed of the anti-TB drug side effects, about 13.0% admitted to not being informed, and 14.0% said that they became aware of the side effects after experiencing them.

#### 3.2.10. Respondents’ View on Obtaining More Information about TB

Respondents were asked to confirm whether they would like to obtain more information about TB. Respondents were asked to choose one of the following three answers: “Yes, No, or I know enough”. About 91.3% wanted more information or education about TB, 7.7% were not interested in obtaining more information about TB, and about 0.97% said that they already had enough information.

### 3.3. Beliefs about TB and Its Treatment

#### 3.3.1. Places Where Respondents Went after Feeling Sick

**Figure 5:**
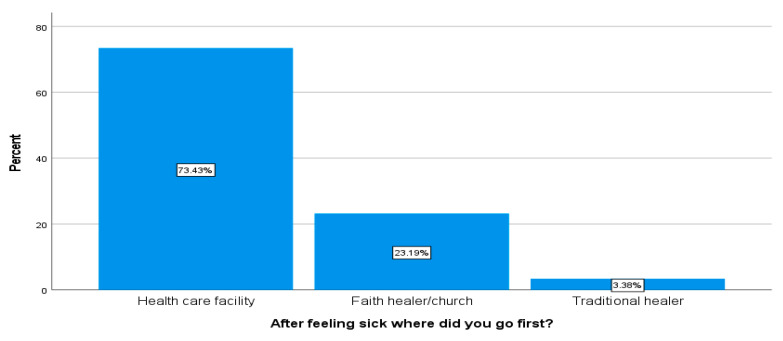
Places where respondents went after feeling sick (*n* = 207).

Respondents were asked to indicate the first place they went for help after feeling sick. Most respondents (73.4%) said that they went to health care facilities, about 23.1% went to faith healers or churches, and 3.3% first went to traditional healers (see Figure 5 below).

#### 3.3.2. Respondents Visited Traditional Healers after TB Diagnosis

Respondents were asked whether they had visited traditional healers after diagnosis. Respondents were asked to choose either “Yes or No”. Most respondents (98.0%) indicated that they had never visited traditional healers, and only 1.9% admitted to having visited traditional healers after being diagnosed with TB.

#### 3.3.3. Respondents Who Visited Faith Healers after TB Diagnosis

Respondents were asked whether they had visited faith healers after their diagnosis, and they were asked to choose one of the following answers of “Yes or No”. A total of 75.3% admitted to having visited faith healers for help after being diagnosed with TB, and about 24.6% had never visited faith healers for help after being diagnosed with TB. 

#### 3.3.4. Exploring Respondents’ Reasons for Visiting Faith Healers or Traditional Healers after TB Diagnosis

The respondents’ reasons for visiting traditional healers or faith healers for help after being diagnosed with TB were explored. Respondents were asked to choose one of the following three answers: “I have strong faith in culture or my religion, I wanted a second opinion about my disease, I do not believe I am sick, or N/A” All respondents (76.8%) who visited traditional healers or faith healers pointed out that they had strong faith in their culture or religion. Respondents (23.1%) who never visited traditional healers or faith healers for help indicated that the question was not applicable to them.

### 3.4. Perception of TB and TB Treatment

#### 3.4.1. Respondents’ Perception about Whether TB Can Be Cured by Traditional Medicines

Respondents were asked whether TB can be treated and cured using traditional medicines. They were asked to choose one of the following answers: “Yes, No, or Don’t know”. Most respondents (92.3%) indicated that TB could not be cured through traditional medicine and 7.7% were not sure whether traditional medicine could cure TB.

#### 3.4.2. Exploring Respondents’ Perceptions for Not Completing TB Treatment

**Figure 6:**
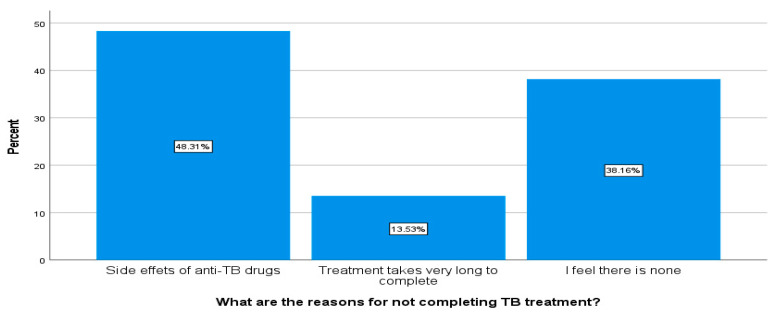
Exploring respondents’ perception for not completing TB treatment (*n* = 207).

The respondents’ perceptions regarding reasons for not completing TB treatment were explored where 48.3% indicated that treatment side effects could make one not complete one’s treatment, 13.5% said that the fact that TB treatment takes a long time can discourage one from completing one’s treatment, and 38.1% said that there is nothing that could cause one not to complete one’s treatment (see Figure 6).

#### 3.4.3. Exploring Whether Respondents Inform Their Families or Friends about Their Appointments at the CHCs

Respondents were asked to indicate whether they had informed their families or friends about their appointment at the CHCs. The answers they had to choose from were “Yes or No”. About 56.5% indicated that they had and 43.4% said that they did not inform them.

#### 3.4.4. Exploring Reasons Why Respondents Do Not Inform Their Families or Friends about Their TB Status or CHC Appointments

The study further explored the reasons why respondents did not inform their families or friends about their TB status or CHC appointments. They were asked to choose one of the following answers: “Fear of being isolated by friends or relatives, No one to trust, or N/A”. A total of 36.2% said that they were afraid of being isolated by their friends or families, 7.2% said that they did not trust anyone, and 56% said that this question was not applicable to them.

## 4. Discussion

The discussion is arranged under sub-headings based on the objectives of the study, namely demographic profile of the respondents, knowledge about TB and TB treatment, beliefs about TB and TB treatment, and perception of TB and TB treatment. The study data show that about 42% of the TB patients were between 18 and 29 years old, 20.8% were between 30 and 39 years old, and 22.2% were between 40 and 49 years old. Most infected people were between 18 and 49 years. This indicates that young economically productive age groups suffer more from tuberculosis. These results are similar to those from previous studies, in which a rapid rise in TB mortality and morbidity among the young adult population between 15 and 44 years of age has been reported [18].

About 93% of the respondents said that they were not aware that they had TB before they consulted the health care facilities and were diagnosed with TB. About 73.9% stated that they only learned about the TB disease after they were diagnosed at the health care facility, and 26% said that they still did not know about TB. Only 65.7% had knowledge on how TB spread from person to person. The study findings revealed that there is still poor TB information in the communities since most respondents only learn about TB when they are already sick. Even after their diagnosis, some respondents still had a poor understanding of TB. Poor knowledge about TB increases misconceptions about the cause and mode of transmission in the community and delays TB diagnosis [19]. Early diagnosis of TB and prompt initiation of treatment are essential for an effective TB control program. Patients with undiagnosed pulmonary TB predominantly act as reservoirs for transmission, and delay in the diagnosis may worsen the disease, increase the risk of death and chances of transmission of TB in the community [20]. 

The current study indicated that about 75.3% respondents had visited faith healers for help even after being diagnosed with TB at the health care facility. Only 3.3% acknowledged that they had visited traditional healers first before going to health care facilities, while 2% acknowledged that they had visited traditional healers even after their diagnosis. Most respondents might be reticent to share intimate information about visiting traditional healers or cultural practices with an outsider who is not related to their culture, therefore, the evidence can be treated with caution [21]. The study further revealed that even though participants acknowledged that TB is caused by a bacteria and that it can be cured if treated correctly, about 76% of respondents indicated that they still had strong beliefs in their culture and religion, and as a result, they acknowledged visiting traditional healers and faith healers for help even after their TB diagnosis as they still linked their disease to a curse or punishment and did not attribute it to a bacteria. This is evident because Limpopo Province has a TB treatment cure rate of 79.9%, which is below the set target of 95%, and a high treatment defaulter rate of 7.4%. This study further revealed that the TB information provided to the TB patients during their health education did not change their perceptions about the TB disease. These findings concur with the findings of a study that was conducted by Gyimah and Dako-Gyeke [22], which revealed that good knowledge of TB treatment practices amongst TB patients did not spontaneously shape perceptions toward TB treatment. A study that was conducted by Viney et al. [23] concluded that most TB patients interviewed in their study did not attribute TB to a bacterial cause. 

Even though most respondents (92.3%) indicated that TB could not be treated with traditional medicine, however, some patients still acknowledged visiting traditional healers for help, which is highly influenced by their cultural orientation. When culture and traditional beliefs oppose the existence of HIV, it becomes difficult for patients who are on treatment to adhere to their treatment [22]. Similarly, Matombo et al. found that cultural practices and beliefs concerning HIV and prevailing faith that traditional healers could treat HIV/AIDS, and the belief that HIV is caused by witchcraft and demons were the factors affecting patients on treatment as patients concurrently visit traditional healers [24]. Another study indicated that some HIV patients stopped taking their ARVs because of cultural influences and lack of family support. Culture plays an important role in the practices of patients on long-term treatments. Patients do not often adhere to treatment because of cultural convictions. Use of herbal or natural remedies was reported before, during, and after TB treatment [25].

The current study shows that about 48.3% perceived that TB treatment side effects causes patients not to complete their treatment. The results of this study showed that a high proportion (59%) of the respondents indicated that they had experienced itchy skin, skin rashes, bruising, and yellow skin, however, those side effects were mostly mild as patients were still taking their treatment during the time of the study. Previous studies have shown that skin rash is very common during the application of first line anti-tuberculosis drugs [26]. The study further revealed that respondents did not want to disclose their TB status to their family members for fear of discrimination and isolation. Due to the lack of comprehensive knowledge about TB treatment, patients were likely to stop treatment. Treatment interruption is also related to perceptions about TB as a disease [27]. Patients also fear being discriminated and isolated within the community as there is a misconception about TB in the community. One study indicated that because of fear of stigma and discrimination, patients did not disclose their HIV status to their family members, which in turn influenced non-adherence to TB medication [28]. 

## 5. Limitation of the Study

The study involved only TB patients on treatment during the time of data collection. Some respondents were not available due to COVID-19 restrictions. Other respondents refused to participate in the study. The study was conducted in a predominately rural province of South Africa. It is likely that if urban areas were included, this could have led to different findings.

## 6. Conclusions

The results of the study highlighted that there is a lack of knowledge, strong beliefs, and incorrect perceptions about TB and its treatment amongst TB patients in the community. Some patients were unaware about the cause of TB and the key routes of its transmission. Poor knowledge about TB in the communities is one of the main risk factors that increase misconceptions and the wrong perception about the TB disease in the community. The lack of knowledge, wrong TB perception, and beliefs about TB disease can lead to delays in TB diagnosis, which in turn can lead to increased TB transmission in the communities. The wrong perceptions about TB can also lead to increased stigma and discrimination in the community, which might encourage the social isolation of TB patients. These findings highlight the need for health education efforts to be implemented to improve community knowledge and awareness of the TB disease by health care providers as it is vital to reduce incorrect perceptions about TB, its transmission, and further promote early TB diagnosis and infection control in the community. 

### Recommendations

Based on the above findings and further COVID-19 concerns, the following recommendations are made:

- The Department of Health should embark on an intensive community education program aimed at changing any wrong perceptions about TB.
- Health care workers should intensify door to door campaigns to improve community TB knowledge.
- Policy makers need to develop guidelines and policies that aim at training traditional healers and religious leaders to be DOT supporters.
- Develop guidelines that encourage family members who provide support to TB patients to be trained on patient supervision.
- Comprehensive COVID-19 and COVID-19 vaccine health education to be provided to TB patients during their visit as people infected with TB may have a weak immune system, may be at a higher risk of contracting COVID-19, and are at a higher risk of suffering more severe COVID-19 symptoms; the risk is even higher if TB patients are co-infected with HIV or diabetes.
- Health care workers need to conduct effective TB screening and testing to differentiate between TB and COVID-19 infection.
- With large number of TB patients who are not diagnosed or treated, it is important for health care workers to conduct effective point-of-care TB screening and make use of new point-of-care TB diagnostic tools to improve TB case identification.

## Figures and Tables

**Table 1:** Demographic profile of the respondents.

Variable		Frequency (*n* = 207)	Percent%
Age	18–29	87	42.0
	30–39	43	20.8
	40–49	46	22.2
	50–59	20	9.7
	over 60	11	5.3
Nationality	South African	174	84.1
	Zimbabwean	33	15.9
Race	African	207	100.0
Marital status	Married	34	16.4
	Single	94	45.4
	Living with a partner	53	25.6
	Widowed	26	12.6
Education level	No schooling	8	3.9
	Primary	69	33.3
	Secondary	98	47.3
	Tertiary	32	15.5
Religion	Christian	194	93.7
	Ancestor	5	2.4
	No religion/others	8	3.9
Occupation	Employed	24	11.6
	Self-employed	38	18.4
	Unemployed	145	70.0
Income	None	136	65.7
	R400–3000	57	27.5
	R3500–15000	9	4.3
	Above 16000	5	2.4

## Data Availability

The data presented in this study are available on request from the first author.

## Supplementary Materials

The following are available online at https://www.mdpi.com/article/10.3390/ijerph181910404/s1, File: ANNEXURE C: TB Patients questionnaire on TB Awareness/Perceptions.
