# Supplementary material for: Knowledge, Beliefs, and Perceptions of TB and Its Treatment amongst TB Patients in the Limpopo Province, South Africa
Source: Int J Environ Res Public Health. 2021 Oct 2;18(19):10404. doi: 10.3390/ijerph181910404 (PMC8508321; doi:10.3390/ijerph181910404)
Supplement: Supplementary file 1 [file ijerph-18-10404-s001.zip › ijerph-1341305-supplementary.pdf]

ANNEXURE C: TB Patients questionnaire on TB Awareness / Perceptions

Title of the Study: Knowledge, beliefs and perceptions of TB and its treatment amongst TB patients in the Limpopo Province, South Africa

Individual Patient's Questionnaire Number: ____________

Date of Interview: ________________________________

INSTRUCTIONS

1. Record the answers by circling a response or by writing the answer in the space provided.
2. Make sure that the participants sign a consent form first before completing this questionnaire.
3. Please note that only one response is allowed per question unless another instruction is given.
4. Please note that coding categories should NOT be read to the participant unless another instruction is given or if participants doesn't understand English and needs assistance.

SECTION A: DEMOGRAPHIC INFORMATION

No	Questions	Coding categories	
A1	What is the name of the area/ village/ township/ town/sub urban that you come from?	………………………………………………………………………………………… ………………………………………… ………………………………………………………………………………………… ………………………	
A2	How do classify yourself in terms of gender?	Male...1<br>Female..2	

A 3	Age	18 to 29 years……………………………………………………………1<br>30 to 39 years………………………………………………………………………2<br>40 to 49 years………………………………………………………………………3<br>50 to 59 years………………………………………………………………………4<br>Over 60 years………………………………………………………………………5	
A 4	What is your nationality?	South African Citizen ....1<br>Mozambican ………………………………………2<br>Zimbabwean ....3<br>Botswana……………………………………………4<br>Non - citizen (permanent resident) ....5<br>Non-citizen (refugee) ....6<br>Other (Specify)...6	
A 5	How do you classify yourself in terms of race?	African ....1<br>White ....2<br>Coloured ....3<br>Indian/Asian...4<br>Other (Specify)...5	
A 6	What is your marital status?	Married ….. …………1<br>Single .... …………2<br>Living with partner<br>……………………………………………………………3<br>Widowed…………………………………………………4<br>Divorced…………………………………………………5	
A 7	What is the highest level of education that you have completed?	No schooling ……………………………………………1<br>Lower primary level……………………………………..2<br>Higher primary level……………………………………3<br>Secondary level………………………..………………4<br>Matric…………………………………………..…………5<br>Tertiary level …………………………………………..6	
A 8	What is your religion	Christian ……………………………………………………………………1<br>Muslim………………………………………………………………………2<br>Ancestors………………………………………………………………3<br>No religion/ other………………………………………………………..4	

A 9	Occupation	Employed.. 1<br>Unemployed.. 2<br>Self-employed... 3	
A 10	What is your income?	R400-R3000…………………………………………………………………………………………………1<br>R3500-R15000…………………………………………………………………………………………………2<br>R16000-and above……………………………………………………………………3<br>None……………………………………………………………………………………………….4	

SECTION B: KNOWLEDGE ABOUT TB AND TB TREATMENT

**No**	**Questions**	**Coding categories**	
B1	When you were sick and had signs and symptoms were you aware that you could be having TB?	Yes ……1<br>No ……..2<br>I was suspicious/ but I was not so sure ……………………………………………………………….…..3	
B2	When did you first hear or learn about tuberculosis or TB	Before I was diagnosed with TB …………………………………………………1<br>During the time I was diagnosed with TB……………………………….……….2<br>After I was diagnosed with TB ……………………………………………………..3	
B3	Do you know what causes TB?	Bacteria……………………………………………………………………………………1<br>Evil eye or witchcraft……………………………………………………..2<br>Don't know……………………………………………………………………………3	

B4	How does TB spread?	Cough (Air) ……………………………………………………………………………………1<br>Unclean food or water / Public area………………………………………….2<br>Hereditary …………………………………………………………………………………….3<br>Sexual contact with TB patient ……………………………………………………4<br>Don’t know ……………………………………………………………………………………5	
B5	TB can be cured if TB treatment is taken daily for the correct treatment duration (6-8 months)	Yes……………………………………………………………………………………………1<br>No………………………………………………………………………………………..2	
B6	Can TB result in death if not treated?	Yes……………………………………………………………………………………………1<br>No………………………………………………………………………………………..2<br>Don’t know………………………………3	
B7	Why is it important to take TB treatment for the prescribed duration (6-8 months)?<br>**(Tick all that applies)**	To prevent drug resistant TB: Yes…….1; No…………..2<br>To be cured: Yes…………….1; No……………………2<br>To prevent the spread of TB: Yes……………….1; No………………2<br>To prevent death: Yes…………..1; No……………..2<br>Other (specify)………………………………………………………………………..	
B8	From the time you started taking treatment have you ever experienced any treatment side effects? And which ones have you experienced, **please choose all that are applicable**.	I have experienced dark coloured urine…………………………………………………1<br>I have experienced Itchy skin, skin rashes, bruising and yellow skin…………………………………………………….2<br>I have experienced lack of feeling or tingling in the hand or feet……………………………………………..3<br>I have experienced upset stomach, nausea, vomiting, diarrhoea, and loss of appetite ……………………………………………..4<br>I have experienced yellow eyes ……………………………………………..5<br>I have experienced none ……………………………………………..6	

B9	During health education were told about TB treatment side effects	Yes ………………………………………………………………………………………………..…1<br>No …………………………………………………………………………………………………….2<br>I was only informed when I had already experience treatment side effects……………………………………………..3	
B10	Were you told of what to do if you experience any of the treatment side effects?	Yes……………………………………………………………………………………………………1<br>No…………………………………………………………………………………………………….2<br>Only when I had experienced them…………………………………………………….3	
B11	Would you like to have more information about TB?	Yes ....1<br>No...2<br>I know enough……………………………………………………………………………3	

SECTION C: CULTURAL, RELIGIOUS AND TRADITIONAL BELIEF

No	Questions	Coding categories	
C1	After feeling sick where did you go first?	Clinic/health care facility or doctor………………………………………………..1<br>Faith healer/church…………………………………………………………….2<br>Traditional healer………………………………………………………………….3	
C2	Did you go to a traditional healer after you were diagnosed with TB?	Yes………………………………………………………………………......................1<br>No…………………………………………………………………………........................2	
C3	Did you go to a faith healer after you were diagnosed with TB?	Yes………………………………………………………………………......................1<br>No…………………………………………………………………………........................2	
C4	Why did you decide to visit a traditional healer or a pastor/prophet/faith healer?	I have strong faith on culture or my religion………………………………1<br>I wanted second opinion about my disease…….2<br>I do not believe I am sick……………………..3<br>N/A……………………………………4	

C5	Were you told the reason why you were sick? And if yes specify? **If this question is not applicable to you, please ignore it.**	………………………………………………………… ……………………………………………… ………………………………………………………… ……………………………………………..	

SECTION D: PERCEPTION OF TB AND TB TREATMENT

No	Questions	Coding categories	
D1	TB can be treated and cured using traditional medicines?	Yes………………………………………………… ……………………………………….1<br>No…………………………………………………… ……………………………………….2<br>I don't know………………………………………………… …………………………..3	
D2	What are the reasons that could make one not completing TB treatment? (Tick all that applies)	Treatment takes very long to complete………………………………………….1<br>Side effects of anti-TB drugs………………………………………………… ……….2<br>Too many pills to take daily: ………………………………………………………… ….3<br>TB can still be cured even if treatment is not completed: ………………...4<br>I feel there is none………………………………………………… ……………………………………………..5	
D3	Did you inform your family of friends that you were on TB treatment and about your clinic visits?	Yes………………………………………………… ……………………………………………1<br>No…………………………………………………… …………………………………………..2	
D4	If no, why?	Fear of being isolated by friends or relatives…………………………………..1<br>No one to trust………………………………………………… …………………………..2<br>N/A…………………………………………………3	
D5	Do you think TB and HIV patients are stigmatized in your community?	Yes………………………………………………… ……………………………………………1<br>No…………………………………………………… …………………………………………..2	
D6	In your community, how are you treated since you were diagnosed TB? **Multiple responses possible**	I feel most people reject me...........................1<br>I feel most people are friendly, but they generally try to avoid me ....2<br>The community mostly supports me ....3<br>Other (Specify)……………………………………………… ………………………………….4	

D8	In your own view do you think the community people have different understanding about TB which is different from what you were told at the clinic? **If so, please specify what people think about TB**	………………………………………………………… ……………………………………….……………………… ………………………………………………………… ……………………..	
